# Philadelphia Hospital

**Published:** 1838-12-19

**Authors:** 


### CLINICAL LECTURE.

### PHILADELPHIA HOSPITAL.

#### ENTROPIUM—PHYMOSIS, &C.

*Saturday*, *December* 8*th*.—Dr. Gibson commenced: Gentlemen, the first case which I shall present to you, to-day, is the one of entropium, upon which you saw me operate two weeks since. This troublesome affection is caused by an inversion of the lid—generally the upper one—by which the eyelashes sweep the cornea, annoying the patient incessantly, and producing a good deal of pain and inflammation. Both patients and surgeons commit a great mistake when they anticipate relief from pulling out the hairs; these are speedily reproduced, and, from being now stumpy and irregular, are a more constant source of irritation than ever. Bear, then, in mind that the cilia are not to be meddled with. There are several operations for this disease. One is, removing with the forceps and a pair of curved scissors, an oval piece of the integument of the eyelid, near to the tarsus; the edges of the wound are then to be united with a fine suture. Another is, removing the whole of the tarsus, and, along with it, the cilia; care being taken not to interfere with the puncta lachrymalia. The removal of the tarsus is generally sufficient to restore the lid to its natural position. This method, however, as well as that of partial removal of the tarsus, is often followed by an unpleasant condition of things, for the gutter for the tears having been destroyed, they run constantly over the cheeks, keeping up incessant epiphora. When the case is a recent one, and not very severe, I prefer the first operation,—the removal of an oval portion of superfluous skin,—and it is the operation which you may remember that I performed on this man. You observe the considerable amendment which has already taken place. The contraction of the cicatrix has had the effect of shortening the lid, and, I think, that in a little while a complete cure will be effected. Should my anticipations, however, not be realized, I shall then perform another simple operation. I shall touch the lid, externally, with a red-hot iron wire. You see this child on the table; its great toe is turned downwards and outwards to such an extent as to prevent, in a great measure, the use of its foot, and, consequently, to impede its walking. This contraction of the toe is permanent, and is the result of a burn; not an unfrequent occurrence in injuries of this description about the joints. The only remedy for this is amputation of the toe, and this I shall probably perform at our next meeting. I might cut through the cicatrix, and restore the toe to its right line, but, most probably, on the healing of the wound, contraction to the same extent would again take place, besides the risk of tetanus the patient would run from the division of nerves and tendons. I show you this case as illustrative of the operation by the *cautery*, and as elucidative of the principle on which it acts. The tendency to contraction in the cicatrix of a bone is very great, and by running a red-hot iron wire along the lid for some distance, I shall produce a similar state of things to that which you see in this boy.

I will now show the operation for *phymosis.* This condition of parts is the result either of original conformation, or of disease—inflammation in syphilis, or gonorrhœa. You are to remember that, so long as any inflammation exists, you are not to attempt an operation for its relief, for it will be followed by severer inflammation, ending, possibly, in mortification, and the patient may lose his penis, and indeed his life. Phymosis is sometimes congenital. The opening in the prepuce is, in some cases, not larger than a hair, and is productive of great inconvenience to the patient. In any attempt to pass the urine it collects between the prepuce and the glans, and forms a pouch, and the water slowly distils through the small aperture; so slowly indeed, that the bladder frequently again fills before this bag is fully evacuated. The remedy is to slit up the prepuce, provided no considerable pouch exists. Concerning this operation, you may ask two questions. One is, where the cut is to be made? and the other, whether the loose edges of the flaps of the prepuce will eventually be rounded off, or whether they will not, in future, prove a source of inconvenience? In answer to the first question, as to the proper spot for the incision, I prefer directly in front, as I invariably find that the flaps contract, and fall into the line behind the corona, and give no further trouble. I shall slit up the prepuce. The prepuce, you are aware, is composed of two folds of skin, which surrounds the glans penis, much in the way that a common double night-cap does the head. Now both folds of membrane are seldom divided at a single stroke of the knife, but require two separate incisions, the inner membrane frequently rolling over the knife, and escaping it the first time. After division, the two membranes are to be tacked together with a stitch of the interrupted suture.

I shall now proceed to operate. I find, upon making the cut, that the inflammation which has produced the phymosis has been so great as to cause the complete adhesion of the two folds. This is not a common occurrence. In this case the suture becomes unnecessary; disease has already anticipated us.

Here is another case of phymosis. You see the glans pressing out a little way; but during erection it must be forced back. In this instance I expect to find a more natural condition of parts, and meet with the double membrane. [Dr. Gibson now operated.] It has turned out as I anticipated. Here are the two folds, and I shall tack these together with the uninterrupted suture to produce adhesion. You observe, on my doing so, how much more natural and better their appearance becomes. It has happened very well that you have had an opportunity of witnessing both varieties of this operation.

Here is a man I showed you sometime since, with an elongated prepuce. He then refused to have any operation performed, as the impediment would, he thought, prove a safeguard to him, or a clog as he termed it. Seeing how well, however, the operation succeeded on the man I circumcised three weeks ago, he has changed his views, and, perhaps, his principles, and is anxious for me to do unto him likewise. The operation I shall perform will be that of circumcision. You see I go cautiously to work, otherwise I might slice off a piece of the glans, and my object is to preserve the little he has left. Now, having removed this piece of foreskin, I shall bring the edges together, and tack them with the interrupted suture, otherwise ulceration, to some extent, might occur. I find, in the portion of prepuce removed, the same induration as in the former case; the cellular tissue is destroyed, and the structure of the part materially changed.

You observe with what remarkable fortitude this man has borne the operation, which is a pretty painful one. I would much rather a patient should cry out than restrain, in this manner, his feelings. The chances of a fortunate issue are much greater. Bad consequences frequently result from a determination on the part of the patient to behave manfully. When I have a capital operation to perform, I generally advise the patient, beforehand, not to suppress his sufferings, but, on the contrary, to give full scope to them. The French surgeons understand this. Some patients do not require this advice, for they begin to cry out the moment the knife touches them, and, very frequently, long before, and they generally fare the better for it.

When Chief Justice M—— was operated on, some years ago, by the late Dr. Physick, I noticed, the moment before the operation, his teeth clenched, and his features rigid. I whispered to him to make no effort to restrain his feelings during the operation, and assigned my reason. He thanked me, and complied by giving vent to his feelings, through the medium of sighs and groans, and suffered less, as he afterwards told me, than he otherwise would have done.

[Dr. Gibson now made some remarks on fractured clavicle, exhibited Desault's apparatus, and called on several members of the class to repeat the application; which was done with much exactness and neatness by several gentlemen. The substance of the remarks were the same as those reported at p. 53. He particularly inculcated the importance of attending to Desault's *principles*, which, he was sure, were mechanically correct, and could not be controverted—said it was immaterial what apparatus was used, provided the indications were fulfilled, and warned the class that he should be strict in his examinations on the subject.]

